# Quantitative proteomics reveals TMOD1-related proteins associated with water balance regulation

**DOI:** 10.1371/journal.pone.0219932

**Published:** 2019-07-24

**Authors:** Wenjun Wang, Dingwen Xu, Lijun Zhong, Wenxi Zhang, Jihong Kang, Jing Zhou, Weibo Ka, Dagong Sun, Xue Xia, Lide Xie, Weijuan Yao

**Affiliations:** 1 Chengde Medical College, Chengde, Hebei Province, China; 2 Hemorheology Center and Department of Physiology and Pathophysiology, School of Basic Medical Sciences, Peking University Health Science Center, Beijing, China; 3 School of Medical Science, Yangzhou Polytechnic College, Yangzhou, Jiangsu Province, China; 4 Medical and Health Analytical Center, Peking University Health Science Center, Beijing, China; 5 Department of Mechanics and Engineering Science, College of Engineering, Peking University, Beijing, China; 6 Department of Medical Physics, School of Foundational Education, Peking University Health Science Center, Beijing, China; 7 Key Laboratory of Biology and Medical Engineering, Guizhou Medical University, Guiyang, Guizhou Province, China; Universita degli Studi di Bari Aldo Moro, ITALY

## Abstract

The distal tubule and collecting duct in kidney regulate water homeostasis. TMOD1 is an actin capping protein that plays an important role in controlling the organization of actin filaments. In this study, we found TMOD1 was specifically expressed in distal tubules and collecting ducts. To investigate the role of TMOD1, we created *Tmod1*^*flox/flox*^ mice and bred them with *Ksp-Cre* mice to generate tubule-specific *Tmod1* knockout mice, *Tmod1*^*flox/flox*^*/Ksp-Cre*^+^ (designated as TFK). As compared with control mice, TFK mice showed oliguria, hyperosmolality urine, and high blood pressure. To determine the mechanisms underlying this phenotype, we performed label-free quantitative proteomics on kidneys of TFK and control mice. Total of 83 proteins were found differentially expressed. Bioinformatic analysis indicated that biological processes, including protein phosphorylation and metabolic process, were involved in TMOD1 regulatory network. Gene set enrichment analysis showed that multiple pathways, such as phosphatidylinositol signaling system and GnRH signaling pathway, were strongly associated with *Tmod1* knockout. Western blot validated the down-regulation of three proteins, TGFBR2, SLC25A11, and MTFP1, in kidneys of TFK mice. Our study provides valuable information on the molecular functions and the regulatory network of *Tmod1* gene in kidney, as well as the new mechanisms for the regulation of water balance.

## Introduction

Actin cytoskeleton regulates cell shape, cell motility, and cellular processes [1]. In kidney, actin and actin related proteins are involved in regulating water and salt homeostasis. There are several channels interact with actin, such as aquaporin 2 (AQP2), cystic fibrosis transmembrane regulator (CFTR), and epithelial sodium channel (ENaC), etc. [2]. As an example, AQP2 is associated with globular actin (G-actin). When phosphorylated, AQP2 disassociates from G-actin and binds to tropomyosin 5b (TM5b), resulting in destabilization of filamentous actin (F-actin) network. Evidence showed that proteins that regulates actin polymerziation, such as Rho GTPase [3], integrin-linked kinase (ILK) [4], and A-kinase anchoring protein 220 (AKAP220) [5], and ezrin [6], participated in AQP2 membrane targeting and endocytosis. Proteomic analysis on the apical membrane of vasopressin-treated mouse cortex collecting duct revealed ~100 significantly changed proteins, 20% of which are associated with actin cytoskeleton organization [7]. This highlights the important roles of actin cytoskeleton in regulation of renal function.

Tropomodulin1 (TMOD1) is an tropomyosin (TM) binding protein and first found in human erythrocytes [8]. By binding with N-terminus of TM, TMOD1 caps the slow-growing end (pointed end) of TM-coated actin filaments (F-actin) [9] and decreases the rate of actin depolymerization [10]. TMOD1 is highly conserved in many species, including human, mouse, chicken, and rat [11]. It is widely expressed in terminal differentiated cells, such as erythrocytes, cardiomyocytes, skeletal muscle cells, and lens fiber cells, and neurons, etc. [12–14]. TMOD1 plays an important role in maintaining the length of F-actin, cytoskeletal structure, and the mechanical properties of cells. The complete knockout of *Tmod1* resulted in the early death of mouse embryos at day 9.5–10.5 because of non-contractile heart tube with disorganized myofibrils, accumulation of mechanically weakened primitive erythroid cells in yolk sac, and failure of primary capillary plexuses remodeling [15, 16].

By using *Tmod1*^*lacZ/+*^ mice, we found that *Tmod*1 also expresses in kidney [17]. Yet the roles of *Tmod1* in renal function remain unclear. Green et al. bred *Tmod1*^+*/lacZ*^ mice with cardiomyocyte-specific *Tmod1* overexpressing transgenic (TOT) mice to obtain *TOT/Tmod1*^*lacZ/lacZ*^ mice [18, 19], which made it possible to study the consequence of *Tmod1* knockout in different cell types and tissues. But unfortunately, this mouse model is not suitable to study the renal function of *Tmod1* since it develops dilated cardiomyopathy, which has abnormal vasopressin production [20]. In present study, we first showed that *Tmod1* was specifically expressed in the distal convoluted tubules and collecting ducts of mouse kidney. Then we created *Tmod1*^*flox/flox*^ mice and bred them with *Ksp-Cre* mice to generate renal tubule and collecting duct-specific *Tmod1* knockout mice, *Tmod1*^*flox/flox*^*/Ksp-Cre*^+^ (designated as TFK). As compared with *Tmod1*^*flox/flox*^*/Ksp-Cre*^−^ mice (designated as TF), TFK mice showed oliguria, hyperosmolality urine, and high blood pressure. To explore the underlying mechanism, we performed quantitative proteomic analysis on the kidneys of TF and TFK mice. Total of 83 proteins were found differentially expressed in TFK mice. Bioinformatic analyses indicated that TMOD1 was closely associated with metabolic process, protein phosphorylation, and multiple signaling pathways. Our study provides valuable information on the molecular functions and the regulatory network of *Tmod1* gene in kidney.

## Materials and methods

All animal procedures were approved by Peking University Committee on Animal Care and Use. The approval number is LA2017095. When needed, the mice were anesthetized by intraperitoneal injection of sodium pentobarbital (50mg/kg) and then sacrificed by cervical dislocation.

### *Tmod1*^+*/lacZ*^ mice and generation of renal tubule-specific *Tmod1* knockout mice

*Tmod1*^+*/lacZ*^ mice were generated in Dr. L. Amy Sung’s laboratory at University of California, San Diego [15] and maintained at Peking University Health Science Center. Cre/loxP system was used to generate the renal tubule-specific *Tmod1* knockout mice. *Tmod1*^*flox/flox*^ mice were created by Cyagen Biosciences Inc. (Santa Clara, CA) by using TetraOne^™^ gene targeting technology. Exon 3, the second coding exon, of *Tmod1* gene was flanked by two floxP sites and both floxP sites were incorporated into mouse genome by homolog recombination. A transgenic mouse line specifically expressing Cre recombinase in renal tubular epithelial cells under the control of *Cadherin 16* (*Ksp*) promoter was purchased from The Jackson Laboratory (Bar Harbor, ME). Female *Tmod1*^*flox/flox*^ mice were bred with male *Ksp-Cre* mice to obtain *Tmod1*^*flox/flox*^*/Ksp-Cre*^+^ mice (designated as TFK mice). The litter mates with *Tmod1*^*flox/flox*^*/Ksp-Cre*^−^ genotype was designated as TF mice and used as control.

### Immunohistochemistry

Kidneys of *Tmod1*^+*/lacZ*^ mice were embedded in paraffin and consecutive sections (5 μm) were cut. After antigen-unmasking and blocking, antibodies of β-gal (MP Biomedical CAPPEL, Irvine, CA), AQP1, Calbindin D-28k, AQP2 (Boster Biological Technology, Wuhan, China), and Tamm-Horsfall protein (THP, Santa Cruz Biotech., Santa Cruz, CA) were applied. HRP-conjugated IgG was added and diaminobenzidine (DAB) solution was used for colorimetric development. The nuclei were counter-stained by hematoxylin staining.

### Microdissection of mouse renal tubules

Mice were anesthetized by intraperitoneal injection of sodium pentobarbital (50mg/kg) and then sacrificed by cervical dislocation. The kidneys were quickly removed and the capsules were pealed off. Several 0.5–1 mm slices were cut from the middle part of the kidneys and then ground on a stainless steel mesh with 0.1 mm pore size. The mesh was washed with PBS and ground tissues were collected into a centrifuge tube. The centrifuge tube was sit for an hour to let the tissues precipitate. The precipitated tissues were transferred into a petri dish and the tubule structures were picked up under dissection microscope with fine-tip forceps.

### Genomic DNA isolation and ploymerase chain reaction (PCR)

Mouse kidney and renal tubules were cut and incubated at 55°C overnight in SNET solution containing 20 mmol/L Tris-HCl (pH8.0), 5 mmol/L EDTA (pH8.0), 400 mmol/L NaCl, 1% SDS, and 200 μg/ml proteinase K. Genomic DNA was extracted by phenol/cholorform/isopropanol (25:24:1) solution. 100 ng genomic DNA was used as template and mixed with 2× Taq PCR mix (Tiangen, Beijing, China), primers, and distilled water. The primer sequences for validating the existence of loxP sites and the deletion of exon 3 were as follows: F2: 5’-CAG CAA ACC ATC CCT GTT CCC AA-3’, F3: 5’-CAC AGA GTG CAA GAG GAT TAG CAA-3’, and R2: 5’-TGA GAG AAG AGA TGA AGG ATT GCC-3’. The primer sequences for identifying the existence of *Cre* gene were 5’-GCA GAT CTG GCT CTC CAA AG-3’ and 5’-AGG CAA ATT TTG GTG TAC GG-3’. The PCR program was 94°C for 5 min, and then 35 cycles of 94°C for 30 s, 65°C for 30 s, and 72°C for 45 s, followed by extension at 72°C for 5 min. The PCR products were separated in 2% agarose gel and visualized by ethidium bromide staining.

### Reverse transcription and quantitative polymerase chain reaction (qPCR)

Total RNA was extracted from kidney or renal tubules by using RNAtrip reagent (Applygen, Beijing, China). 2 μg of RNA was reverse transcribed into cDNA. Then cDNA was mixed with primers, Brilliant II SYBR Green QPCR Master Mix (Yeasen, Shanghai, China) and RNase-free H_2_O and qPCR was performed on a Mx3000 Multiplex Quantitative PCR system (Stratagene). The primers used for *Tmod1* were 5’-GACACAGCCTCACACAATGT-3’ and 5’-CTTGGTGGTCTGATCCTTCT-3’. *Gapdh* primers were used for the internal control. The sequence were 5’-ACCACAGTCCATGCCATCAC-3’ and 5’-TCCACCACCCTGTTGCTGTA-3’. A relative fold change in gene expression was calculated using the method of 2^-ΔΔCt^.

### Western blot

Total proteins were extracted from kidney and renal tubules with RIPA buffer. 100 μg protein were separated by SDS-PAGE and transferred to PVDF membrane. The membrane was blocked at room temperature for 1 h in TBST (Tris buffered saline containing 0.1% Tween 20) containing 5% skimmed milk and was then incubated with primary antibodies at 4°C overnight. The membrane was washed for 5 min with TBST buffer for 3–5 times and then incubated with a horseradish peroxide-conjugated secondary antibody at a dilution 1:4000 at room temperature for 1 h. After washing, the membrane was developed with ECL Reagent and exposed to Kodak XBT-1 film. Anti-TMOD1 antibody was prepared by AbMax Biotechnology Co., Ltd. (Beijing, China). Anti-GAPDH antibody was purchased from Santa Cruz Biotech. (Santa Cruz, CA). Antibodies for TGFBR2, SLC25A11, MTFP1 were from Abclonal (Wuhan, China).

### Metabolic cage analysis

Mice were placed in metabolic cages to acclimate for 24 hours. For basal level test, mice had free access to water and food and 24-hour urine was collected. The urine osmolality was measured with an osmometer (Advanced Instruments, Inc., Norwood, MA).

### Blood pressure test

The systolic blood pressure was measured using the tail-cuff method. The mice were kept awake and their blood pressures were measured in 5 consecutive days. The first two days were for adaptation and the data obtained in the last 3 days were used for analysis.

### AVP measurement

Plasma AVP was measured using Arg^8^-Vasopressin ELISA kit (Enzo Life Sciences, Farmingdale, NY).

### Sample preparation for shotgun proteomic analysis

PBS perfused kidneys were homogenized in RIPA lysis buffer (supplemented with 1mmol/L PMSF) on ice. The homogenates were placed on ice for 10 min and centrifuged at 12,000 rpm and 4°C for 5 min. The supernatant was collected and boiled at 100°C for 5 min. The concentration of total proteins was measured using bicinchoninic acid assay kit (Applygen, Beijing, China). Then proteins samples (200 μg) in triplicates were processed for filter-aided sample preparation (FASP) according to the manufactures protocol. In brief, protein samples were mixed with 100 μL of 8 mol/L urea in 0.1 mol/L Tris/HCl (pH 8.5) and then centrifuged at 14,000×*g* at 20°C for 15 min. The procedure was repeated once. 10 μL of 0.05 mol/L Tris-(2-carboxyethyl) phosphine was added onto the filters and incubated at 37°C for 1h. Then 10 μL of 0.1 mol/L iodoacetamide was added and the filters were kept in dark for 30 min. After centrifugation, filters were washed twice with 200 μL of 50 mmol/L NH_4_HCO_3_ solution. Then 100 μL of NH_4_HCO_3_ solution with 4 μg tripsin (enzyme to protein ratio 1:50) was added onto filters and incubated overnight at 37°C. Finally, the released peptides were collected by centrifugation. The digested peptide mixtures were reconstituted in 20 mmol/L mammonium formate (pH10) and eluted by high pH reverse chromatography using the Dionex Ultimate 3000 Micro Binary HPLC Pump System. The eluted peptides were pooled and vacuum dried.

### Liquid chromatography-tandem mass spectrometry (LC-MS/MS)

The MS analyses were performed on a nano-flow HPLC system (Easy-nLC II, Thermo Fisher Scientific, Waltham, MA) connected to a LTQ-Orbitrap Velos Pro (Linear quadrupole ion trap-Orbitrap mass analyser) mass spectrometer (Thermo Fisher Scientific), equipped with a Nanospray Flex Ion Source (Thermo Fisher Scientific). The published procedures were followed [21]. All samples were analyzed in triplicates.

For protein identification, MS/MS data were submitted to Uniport human protein database (release 3.43) using Andromeda search engine [21]. The identified proteins were imported into a Microsoft excel file for further analysis. Label-free quantitation (LFQ) was performed by using MaxLFQ algorithm in MaxQuant software suite (version 1.4.1.2). MaxLFQ algorithms use “delayed normalization” and “maximal peptide ratio extraction” to achieve accurate proteome-wide label-free quantification [22]. The minimum ratio count for LFQ was set to 2, and the match-between-runs option was enabled. Perseus software (version 1.4.1.3) was used for statistical analysis of the MaxQuant output. A twofold change and *p*<0.05 were used as combined thresholds to define differentially expressed proteins.

### Bioinformatic analyses

The hierarchical cluster and heatmap analyses on the differentially expressed proteins were carried out in HemI (Heatmap Illustrator, version 1.0.1, http://hemi.biocuckoo.org/down.php) [23]. The Database for Annotation, Visualization and Integrated Discovery analysis (DAVID) Bioinformatics Resources 6.8 (https://david.ncifcrf.gov/) was used to provide functional annotations for the significantly differentially expressed genes [24, 25], which included the Kyoto Encyclopedia of Genes and Genomes (KEGG) pathway analysis and Gene Ontology (GO) analysis. Terms of GO_Biological Process (GO_BP), Cellular Component (GO_CC), and Molecular Function (GO_MF) were obtained and those with *p*-value <0.05 were considered as the significantly enriched terms.

The BisoGenet plugin in Cytoscape 3.6 environment was used to expand the protein interaction network without *Tmod1* gene [26]. The significantly differentially expressed proteins were used as queries and hyperlinks, including BIOGRID, DIP, BIND, MINT, and INTACT, were connected to retrieve the protein interaction networks. Then BiNGO plugin in Cytoscape environment was used to retrieve the Gene Ontology Consortium for all the genes in the interaction network. Gene set enrichment analysis (GSEA) was used to analyze all the proteins with more than two unique peptides from LFQ intensity instead of selecting significantly differentially expressed proteins [21, 27, 28]. The phenotypes of analyzed data were given to two classes, A (TFK) and B (TF). All curated canonical pathways (C2) in KEGG pathway database (v.6.2) were selected as the gene sets. The permutation type was set to gene set. The pathways with nominal *p*-value *<* 0.05 and FDR *<* 0.25 were considered as significantly enriched pathways. Then the enriched pathways were subjected to Cytoscope and interpreted using Enrichment Map plugin.

### Statistical analysis

Results were expressed as mean ± standard error of the mean (SEM). Statistical evaluation was performed with the Student’s *t*-test when two value sets were compared. *p <* 0.05 was considered to be significant.

## Results

### TMOD1 is localized in distal tubule and collecting duct in kidney

**Fig 1:**
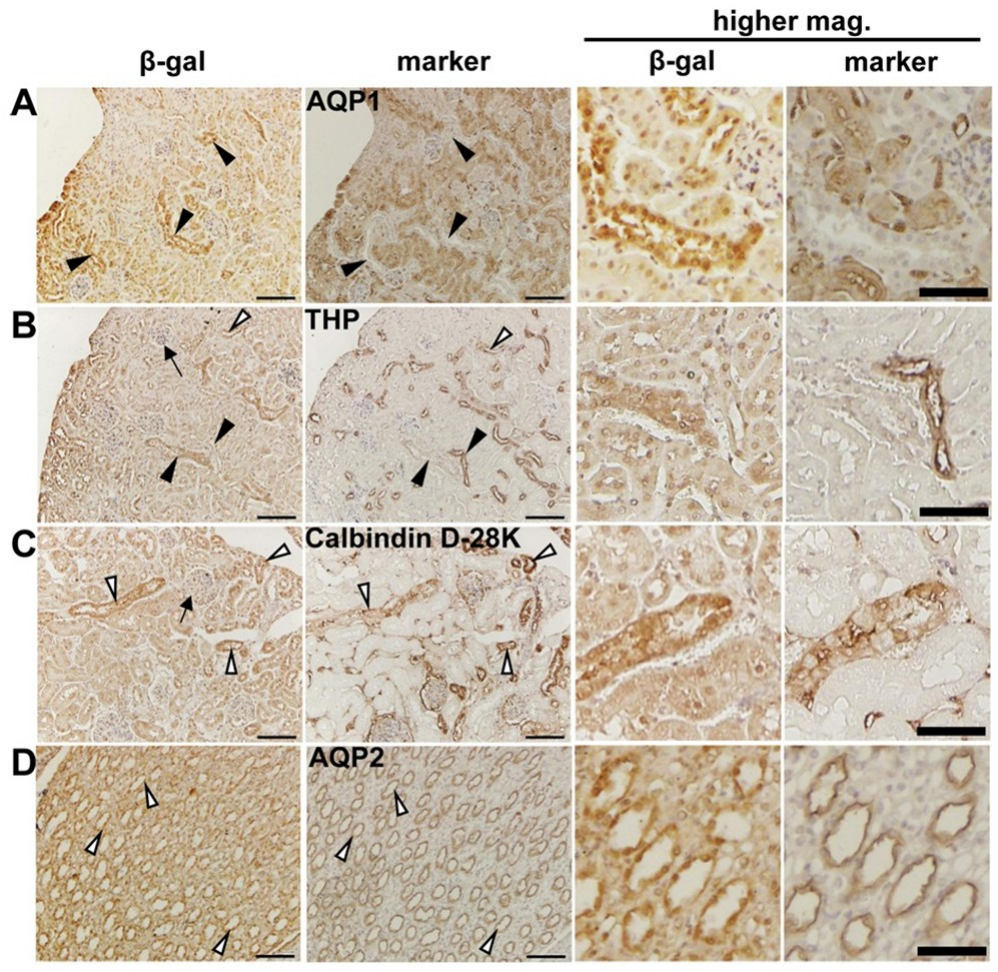
The localization of TMOD1 in mouse kidney. Immunohistochemistry was performed on the consecutive renal sections of *Tmod1*^+*/lacZ*^ mice using anti-β-gal antibody (indicating TMOD1) and antibodies for markers of different tubular segments. (A) AQP1 for proximal tubule; (B) THP for ascending thick limb of Henle’s loop; (C) Calbindin D-28K for distal convoluted tubule; (D) AQP2 for collecting ducts. White and black arrow heads indicates positive and negative colocalization of β-gal and the markers, respectively. Arrows in (B) and (C) points to a glomeruli. Images with higher magnification were shown on the right panels. Scale bars: 100 μm (for lower mag. images) and 50 μm (for higher mag. images).

We previously showed that *Tmod1* is expressed in kidney and localized in some tublular structures [17]. To find out the exact structures expressing TMOD1, we performed immunohistology on consecutive tissue sections of kidneys of *Tmod1*^+*/lacZ*^ mice. In *Tmod1*^+*/lacZ*^ mouse, knocked-in *lacZ* gene is under the control of *Tmod1* promoter, thus its product, β-galatosidase, indicates where *Tmod1* is expressed. Antibodies for AQP1, THP, Calbindin D-28K, and AQP2 were applied to indicate proximal tubule, thick ascending limb, distal tubule, and collecting duct, respectively. Results (Fig 1A–1D) showed that β-gal signals were colocalized with Calbindin D-28K and AQP2, but not with AQP1. There was occasional colocalization between β-gal and THP. At the same time, β-gal staining was negative in glomeruli. The data suggest that TMOD1 specifically expresses in distal tubule and collecting duct in kidney.

### Construction and validation of renal tubule-specific *Tmod1* knockout mice

**Fig 2:**
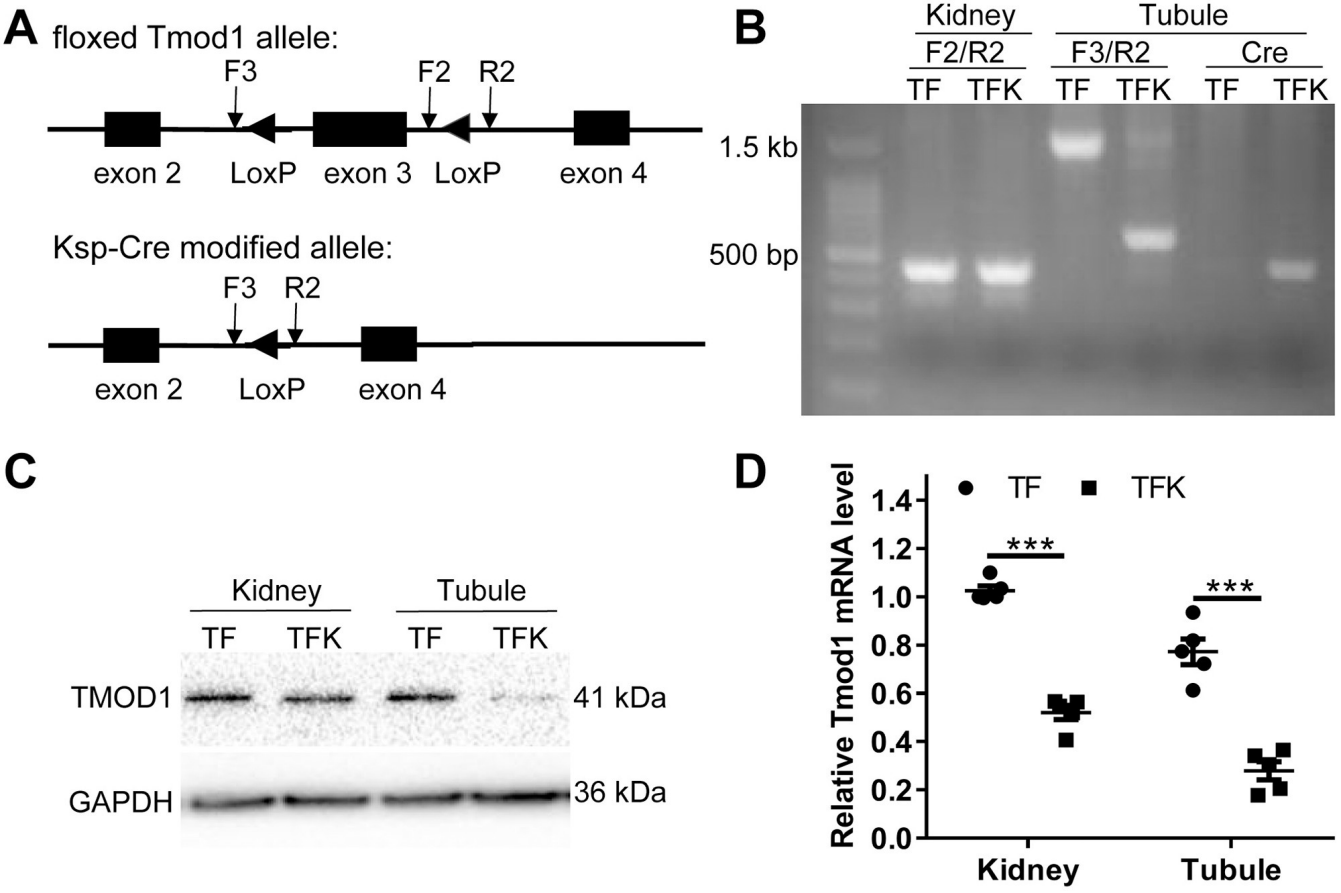
The construction and validation of renal tubule-specific *Tmod1* knockout mouse model. (A) Schematic diagram showing gene structures of the floxed *Tmod1* allele and *Ksp-Cre* modified allele. LoxP sites were inserted flanking exon 3 of *Tmod1* gene. The expression of Cre recombinase in renal tubules results in the excision of exon 3. The positions of genotyping primers, F2, F3 and R2, were marked in the diagram. (B) Genotyping of renal tubule-specific *Tmod1* knockout mice by PCR. The genomic DNAs were isolated from kidneys and manually isolated tubules of *Tmod1*^*flox/flox*^*/Ksp-Cre*^+^ (TFK) and *Tmod1*^*flox/flox*^*/Ksp-Cre*^−^ (TF) mice. Primer sets, F2/R2, F3/R2 and Cre primers were used and their corresponding PCR products were 461 bp, 617 bp (in TFK) or 1.5 kb (in TF), and 420 bp, respectively. The products were separated in agarose gel. (C-D) Validation of renal tubule-specific Tmod1 knockout mice. Protein or RNA were isolated from kidneys and and manually isolated tubules of TF and TFK mice. The expression of TMOD1 was detected by Western blot (C) or qPCR (D). GAPDH was used an internal control. ***: *p* < 0.001.

To study the role of *Tmod1* in kidney, we first created *Tmod1*^*flox/flox*^ mice by inserting floxP sites flanking exon 3 of *Tmod1* gene (as illustrated in Fig 2A). *Ksp-Cre* transgenic mice, in which Cre recombinase expression is limited to epithelial cells of renal tubules, especially the loops of Henle, distal tubules, and collecting ducts [29], were used to breed with *Tmod1*^*flox/flox*^ mice. *Tmod1*^*flox/flox*^*/Ksp-Cre*^+^ mice (TFK) and *Tmod1*^*flox/flox*^*/Ksp-Cre*^−^ mice (TF) were obtained. PCR using genomic DNA isolated from whole kidneys and F2/R2 primer set generated a band of 461 bp in both TF and TFK mice (Fig 2B). When using genomic DNA isolated from renal tubules and F3/R2 primer set, a band of 1.5 kb was generated in TF mice, but the band in TFK mice was 617 bp, suggesting the knockout of *Tmod1* gene. We also isolated proteins and RNA from whole kidney and renal tubules of TF and TFK mice. Western blot and qPCR data showed that both TF and TFK kidneys had *Tmod1* expression, but the expression level of *Tmod1* in TFK kidney was about half of that in TF kidney (Fig 2C and 2D). In renal tubules, *Tmod1* was readily detected in TF mice but it was hardly seen or significantly dropped in TFK mice. These suggest the specific knockout of *Tmod1* in renal tubules was successful.

### *Tmod1*^*flox/flox*^*/Ksp-Cre*^+^ mice showed oliguria and high blood pressure

**Fig 3:**
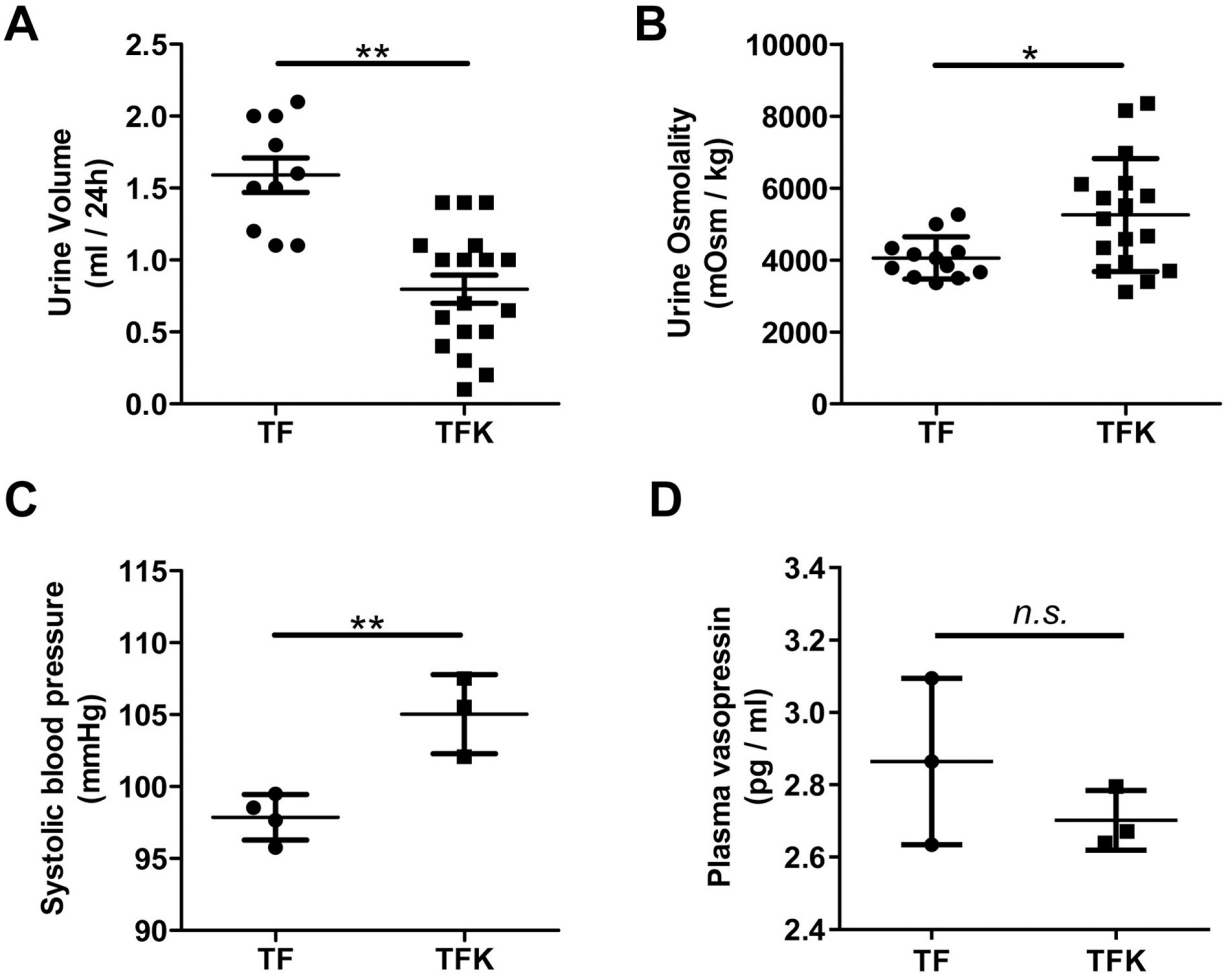
Renal tubule-specific *Tmod1* knockout mouse is characterized with oliguria and high blood pressure. (A-B) TF and TFK mice were subjected to metabolic cage analysis under the basal condition. The urine volume of 24 h (A) and the urine osmotality (B) were obtained. The systolic blood pressures were measured for the mice (C). The plasma vasopressin concentrations were measured with a ELISA kit (D). *: *p* < 0.05, **: *p* < 0.01, *n*.*s*.: no significance.

Next, we sought to examine the effect of *Tmod1* knockout on renal function. TF and TFK mice were placed in metabolic cages and the 24 h urine volume under basal condition was measured. We found that the urine volume of TFK mice was 0.8±0.1 ml, which is much less than that of TF mice (1.6±0.1 ml) (*p*<0.01, Fig 3A). The urine osmolality of TFK mice was 5494±259 mOsm/kg, much higher than that of TF mice (3843±106 mOsm/kg) (*p*<0.05, Fig 3B). These data suggest that TFK mice showed oliguria and hyperosmolality urine, i.e., water retention occurred. Consistently, blood pressure measurement showed that TFK mice had much higher systolic pressure than TF mice (105.0±1.6 mmHg v.s. 97.9±0.8 mmHg) (Fig 3C). Since vasopressin secretion determines the water balance in mice, we measured vasopressin levels in plasma of TF and TFK mice under basal condition. Data showed there was no difference in vasopressin concentrations between TF and TFK mice (Fig 3D), suggesting that water retention seen in TFK mice was not due to abnormal vasopressin secretion.

### Proteomic analysis on kidneys of *Tmod1*^*flox/flox*^*/Ksp-Cre*^+^ mice

**Fig 4:**
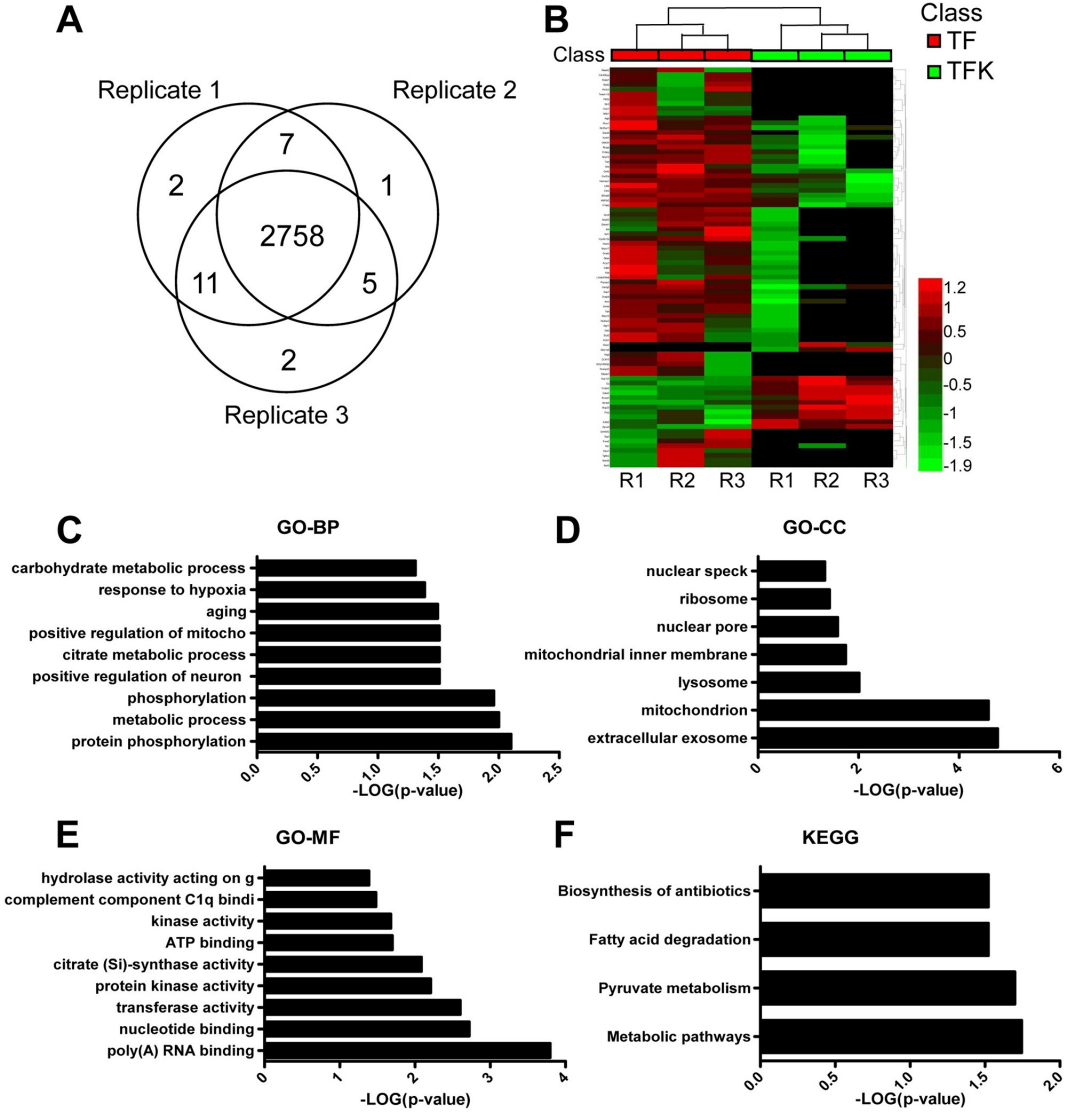
Proteomic study of renal-tubule specific *Tmod1* knockout mouse model. (A) Venn diagram of the identified protein numbers in each of the three biological replicates. (B) Heat map of the 83 differentially expressed proteins in TF and TFK mice. The red color indicates high abundance and green color indicates low abundance. (C-F) Functional annotation of 83 differentially expressed proteins by DAVID Bioformatic Resource. Significantly enriched terms of GO_BP (C), GO_CC (D), GO_MF (E), and KEGG pathways (F) were shown.

Then we aimed to find out the mechanisms for TMOD1’s role in water balance by proteomics. The shotgun proteomic analyses on kidneys of TF and TFK mice identified a total of 4852 proteins, among which 2798 proteins with at least two unique peptides were quantified by the MaxLFQ algorithm. Venn diagram was drawn to show the number of proteins identified in three biological repeats (Fig 4A). All LC-MS/MS data were deposit at MassIVE at University of California, San Diego (https://massive.ucsd.edu/ProteoSAFe/static/massive.jsp). The URL to our dataset (doi:10.25345/C5H63C) is ftp://massive.ucsd.edu//MSV000083953. Perseus software was used to log-transformed (base 2) the raw abundance of proteins to obtain a normal distribution before the differentially expressed proteins were identified. By using the criteria of foldchange > 2 or <0.5 and *p*-value <0.05, we identified 83 significantly differentially expressed proteins. The significantly up-regulated and down-regulated proteins were listed in S1 and S2 Tables, respectively. The expression levels of all 83 proteins in all samples were subjected to hierarchical cluster analysis and shown in a heatmap (Fig 4B). The heatmap clearly showed the difference of protein expression between TF and TFK mice. We also performed functional annotation analysis on these 83 dysregulated proteins by DAVID Bioinformatics Resource. Results of GO analysis showed that protein phosphorylation and metabolic process were the most enriched GO_BP terms (Fig 4C). Extracellular exosome and mitochondrion were the most enriched GO_CC terms (Fig 4D). Poly(A) RNA binding, nucleotide binding, transferase activity, and protein kinase activity were the most enriched GO_MF terms (Fig 4E). KEGG analysis indicated that the dysregulated proteins were highly related to metabolic pathways, pyruvate metabolism, and fatty acid degradation (Fig 4F).

### Protein-protein interaction network construction and gene ontology analysis

**Fig 5:**
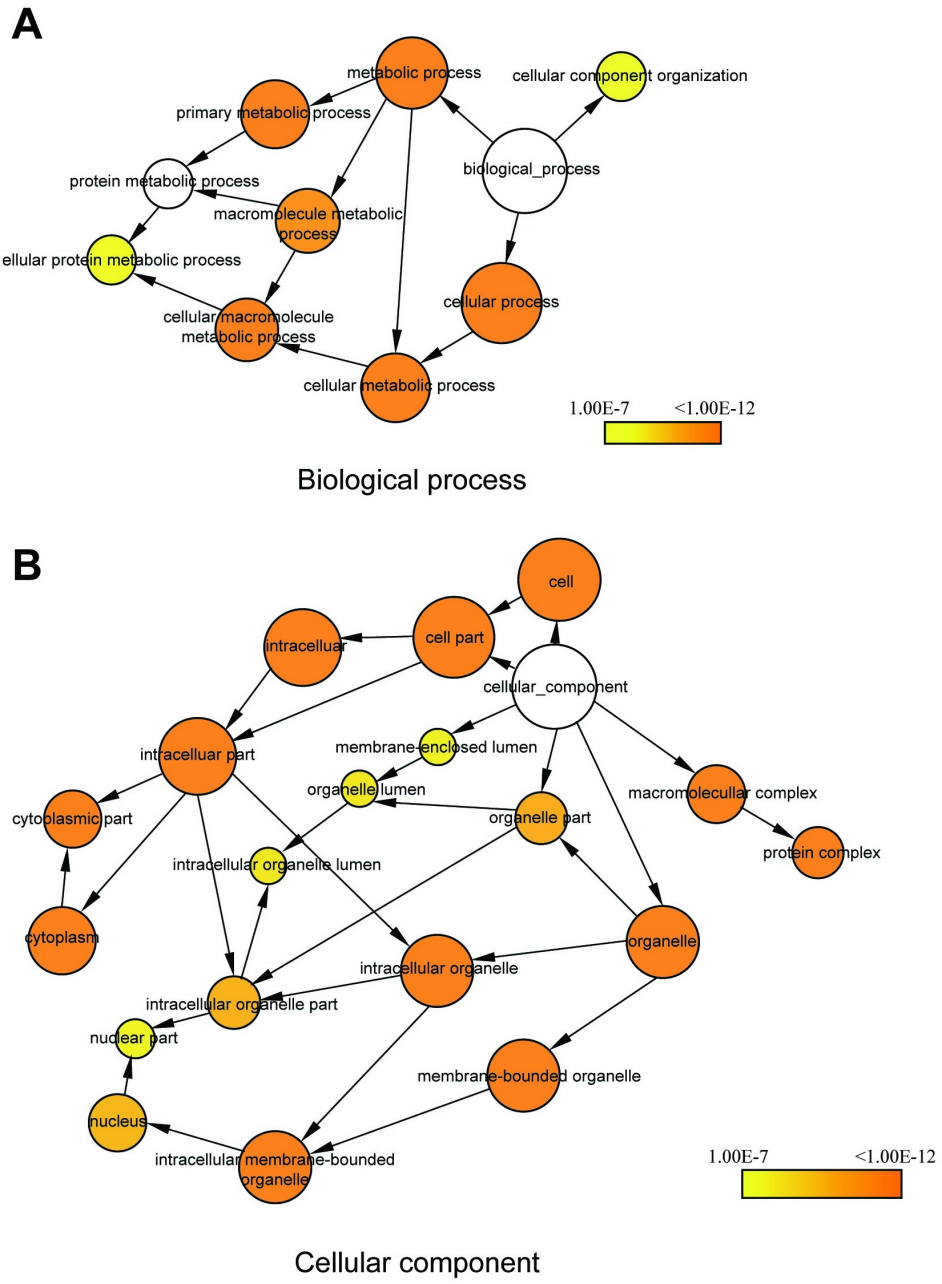
Gene ontology analysis of TMOD1 regulation network. Protein-protein interaction network was constructed using 83 dysregulated proteins in BisoGenet plugin of Cytoscape (v.3.6) and GO analysis were performed on the network using BiNGO plugin. The clusters of biological process (A) and cellular component (B) were shown. The color gradient of the cluster distribution network stands for the *p*-value of each cluster associated with the term. Darker (orange) color indicates a lower *p*-value.

To obtain the protein-protein interaction network associated with all 83 dysregulated proteins, we used BisoGenet plugin in Cytoscape 3.6 environment to link these proteins with several public databases [30]. The resulting network provided 456 nodes and 2177 relationships (S1 Fig). To gain more knowledge on the protein interaction network, the entire gene set in this network was subjected to functional annotation using BiNGO plugin to determine which gene ontology terms were significantly overrespresented [31]. Results showed that the proteins in interaction network were highly correlated with biological processes, such as metabolic process, and macromolecule metabolic process, etc (Fig 5A). They were also highly associated with cellular components, like intracellular part, macromolecular complex, and intracellular membrane bound organelles (Fig 5B). These suggest that TMOD1 is involved in the regulations of these biological processes and cellular components.

### Gene set enrichment analysis

**Fig 6:**
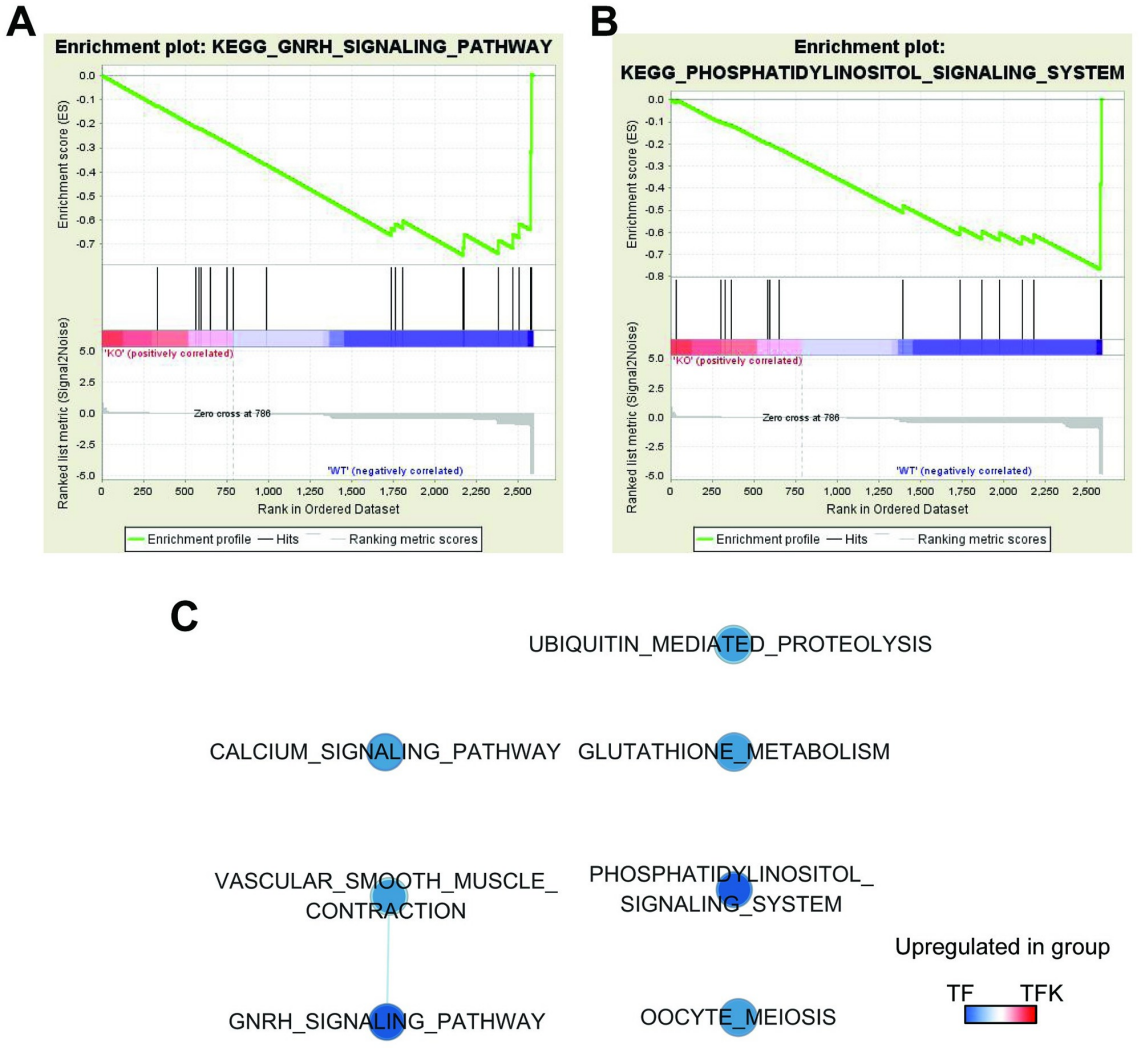
Gene set enrichment analysis (GSEA) on all the identified proteins in TF and TFK mice. (A-B) The enrichment plots (profile of the running ES Score & positions of gene set members on the rank ordered list) for gene sets, GnRH signaling pathway (A) and phosphatidylinositol signaling system (B), were shown. (C) The enrichment map for 7 enriched gene sets at FDR < 50% and nominal *p*-value < 0.05 was drawn in Cytoscape. The blue color of nodes represents the pathways upregulated in TF mice.

In addition to the bioinformatic analyses on significantly differentially expressed proteins and their interacting proteins, we also performed Gene Set Enrichment Analysis (GSEA) on all the proteins that were identified in both TF and TFK mice. This would provide us more understanding on the gene expression changes resulted from *Tmod1* knockout in kidney. The results showed that 2 gene sets in phenotype B (TF group) were significantly enriched at FDR<25% and nominal *p*-value<0.05. They are GnRH signaling pathway and phosphatidylinositol signaling system. The enrichment plots (profile of the running ES Score & positions of gene set members on the rank ordered list) were shown in Fig 6A and 6B. There were 5 more gene sets that were upregulated in TF group at nominal *p*-value<0.05 and FDR <50%. A network of gene sets were constructed to visualize their relation (Fig 6C). The details of 7 gene sets, including name, size, NES, nominal *p*-value, and FDR *q*-value, were listed in S3 Table.

### Verification of differentially expressed proteins by Western blot

**Fig 7:**
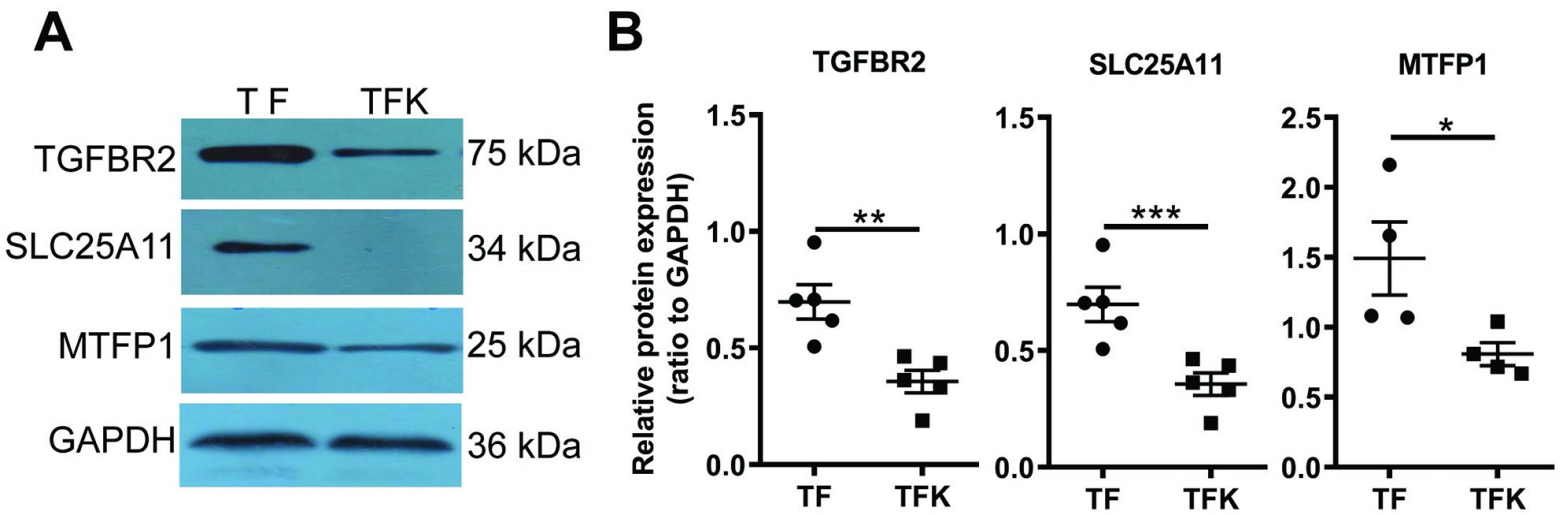
Western blot validation of differential expression of TGFBR2, SLC25A11, and MTFP1. (A) Western blot results showing the expression levels of TGFBR2, SLC25A11, and MTFP1 in TF and TFK mice. GAPDH was used as an internal control. (B) Statistical analysis on the semi-quantification data of bands. Each dot or square represents one replicate. *: *p* < 0.05, **: *p* < 0.01, ***: *p* < 0.001.

We chose three differentially expressed proteins identified by proteomic analysis and validate their expression levels by Western blot. Three proteins are transforming growth factor, beta receptor II (TGFBR2), solute carrier family 25 (mitochondrial carrier oxoglutarate carrier), member 11 (SLC25A11), and mitochondrial fission process 1 (MTFP1), all playing important roles in regulating protein kinase activity, metabolic process, and mitochondrial functions. Western blot data showed that, as compared to TF mice, the expression levels of TGFBR2, SLC25A11, and MTFP1 were significantly reduced in TFK mice (Fig 7A and 7B). This suggests that the proteomic data and bioinformatic analyses were reliable.

## Discussion

In kidney, the distal tubule and collecting duct are critical segments for regulating water balance. The restricted expression of TMOD1 in these structures (Fig 1) suggests that TMOD1 might be involved in regulating water balance of body. Since the existing *Tmod1* knockout mouse model is not suitable for studying TMOD1’s renal function, we generated renal tubule and collecting duct-specific *Tmod1* knockout mice. The successful knockout of *Tmod1* was confirmed by PCR from genomic DNA and Western blot, in which *Tmod1* was barely seen in manually isolated tubules of TFK mice (Fig 2B and 2C). But qPCR data suggested that *Tmod1* was not completely absent in renal tubules of TFK mice (Fig 2D). This discrepancy may come from the different sensitivity of detection methods. Any contamination from other *Tmod1*-expressing tissues, such as blood vessels, would affect the qPCR results. Despite that, TFK mice exhibited distinct phenotypes of oliguria, hyperosmolality urine, and high blood pressure (Fig 3), suggesting water retention occurred. Since the plasma vasopressin level was not altered in TFK mice, their abnormal water retention indicates that TMOD1 regulates the functions of renal tubule and collecting duct via non-vasopressin dependent mechanisms.

To explore the underlying mechanisms for TMOD1’s renal function, we performed LC-MS based proteomic analyses on the kidney of TFK mice, using TF mice as control. Since proteins are essential for the progression of biological processes, proteomic study is useful and important for revealing the functions of specific genes. MS-based proteomics has been significantly improved and LC-MS enables the simultaneous and quantitative analysis on thousands of proteins from complex biological samples [32]. Therefore, proteomics in combination with bioinformatic analysis could provide us important information to understand complex pathogenesis in mouse models [30]. The usage of kidney rather than renal tubules in LC-MS analysis in our study allowed us to examine the changes of proteins in whole kidney in response of *Tmod1* specific knockout.

Total of 83 proteins were found significantly differentially expressed in kidneys of TFK mice (S1 and S2 Tables). DAVID Bioinformatic analysis indicated that these proteins were mainly involved in protein phosphorylation and metabolic process. The most enriched molecular functions were poly(A) RNA binding, transferase activity and protein kinase activity (Fig 4). The construction of protein interaction network for 83 dysregulated proteins and GO analysis using BiNGO plugin on the network provided us a whole picture of functional changes associated with *Tmod1* knockout (Fig 5). The proteins in the network are highly related to metabolic process and intracellular part and organelle. This is consistent with the result of KEGG analysis, in which metabolic pathways were highly enriched (Fig 4F).

The regulation of water balance in renal tubule and collecting duct relies on the expression and membrane localization of water and ion channels. Surprisingly, *Tmod1* knockout did not affect the expressions of any major channel proteins, such as AQP1-4, ENaC, Na^+^-K^+^-2Cl^-^ contransporter, etc., but rather affected the proteins involved in phosphorylation and metabolic process. How would these proteins be related to water balance? We noticed that many phosphorylation related proteins, e.g., PTK2B, RRAGA, PI3K3C3, were the key components of important signaling pathways, such as calcium signaling, mTOR signaling, and PI3K signaling [33–35]. All these signaling pathways have been shown to regulate the dynamics and reorganization of actin cytoskeleton [35–37]. Furthermore, TGFBR2, a receptor for TGF-β, regulates gene transcriptions by phosphorylating SMAD and p38 MAPK and induces actin polymerization by activating Rho-like GTPase pathways [38, 39]. Researchers showed that TGF-β regulated the expression and subcellular localization of an important chloride channel, CFTR, and represents a key regulator of fluid movement [40]. Therefore, it is possible that TMOD1 could induce actin cytoskeleton reorganization through multiple signaling pathways. This may alter the trafficking and cellular localization of channel proteins, which will lead to the change in water and salt reabsorption. It should be pointed out that the knockout of *Tmod1* may directly reduce actin polymerization, while down-regulation of TGFBR2, PTK2B, RRAGA, and PI3K3C3 proteins in TFK mice would further deteriorate this situation. That means TMOD1 may be within or parallel to these signaling pathways in terms of regulating actin cytoskeleton structure.

A number of dysregulated proteins, e.g., NCEH1, ALDH3A2, EHHADH, etc., are closely related to metabolic processes such as lipid, fatty acid, amino acid metabolisms and glycolysis. MCUR1, MRPL15, and SLC25A11 proteins are involved in regulating mitochondria morphology and function. The down-regulation of all these proteins would decrease the metabolic and energy states of epithelial cells in renal tubule and collecting duct of TFK mice, resulting in less ATP production. That may greatly reduce the activities of ATP-dependent ion channels [41, 42] and interfere water reabsorption. In addition, proteins like GALC, GNS, etc., are associated with cellular components, e.g., lysosome and extracellular exosome. PI3K3C3 was shown to regulate Rab7 and late endocytic trafficking [43]. Their down-regulation may hamper the trafficking and endocytosis of channel proteins and affect the water and salt reabsorption.

In order to avoid the bias caused by differentially expressed proteins, GSEA analysis was performed using all identified proteins. Data showed that *Tmod1* deficiency significantly influenced two signaling pathways: GnRH signaling pathway and phosphatidylinositol signaling system (Fig 6). GnRH acts through GnRH receptor and activates multiple signaling pathways inside the cells [44]. Phosphorylated forms of phosphatidylinositol (PI) play important roles in lipid signaling, endocytosis, and vesicle trafficking in kidney [45, 46]. The validated protein, MTFP1 (Fig 7), is a downstream target of PI3-kinase pathway and determines mitochondrial morphology and apoptosis [47]. These indicate that TMOD1 is associated with multiple signaling pathways and regulates different aspects of cell functions. This could be the underlying mechanism for the oliguria phenotype of TFK mice.

Next, one would ask, as an actin capping protein, how does TMOD1 affect the expressions of dysregulated proteins? Actually, in recent years, actin has been recognized as an important regulator of gene transcription [48, 49]. Since TMOD1 protein possesses nuclear exporting signal and nuclear localization signal [50], it would be able to regulate the polymerization state of both cytoplasmic and nuclear actin and alter the transcriptions of many genes. It is also possible that TMOD1 could affect the endocytosis and turnover of some receptors, for example, TGFBR2 and renin receptor, by changing actin cytoskeleton organization. More research is needed to provide evidence for these speculations.

In conclusion, our study presented evidence showing the specific expression of TMOD1 in distal tubule and collecting duct of mouse kidney. By constructing renal tubule-specific *Tmod1* knockout mouse model, we found TMOD1’s renal function is associated with water balance regulation. By proteomic and bioinformatic analyses, we found that TMOD1 was closely related to metabolic process, protein phosphorylation, and multiple signaling pathways. Our results indicate the critical role of TMOD1 in renal function and provide new molecular mechanisms for the regulation of water balance.

## Supporting information

S1 Fig Protein-protein interaction regulatory network construction of the TMOD1 regulation network. The protein-protein interaction network associated with TMOD1 was generated using the BisoGenet plugin of Cytoscape (v.3.6). The red nodes represent input proteins. Blue nodes represent neighbour proteins that have known relationship with input proteins.
(PDF)

S1 Table Significantly up-regulated proteins identified by LC-MS/MS. (DOC)

S2 Table Significantly down-regulated proteins identified by LC-MS/MS. (DOC)

S3 Table Details of the gene sets enriched by GSEA in TF group. (DOC)
