# Supplementary figures and images for: Quantitative proteomics reveals TMOD1-related proteins associated with water balance regulation
Source: PLoS One. 2019 Jul 24;14(7):e0219932. doi: 10.1371/journal.pone.0219932 (PMC6656345; doi:10.1371/journal.pone.0219932)

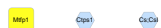

Supplement: S1 Fig — The protein-protein interaction network associated with TMOD1 was generated using the BisoGenet plugin of Cytoscape (v.3.6). The red nodes represent input proteins. Blue nodes represent neighbour proteins that have known relationship with input proteins. (PDF) [file pone.0219932.s001.pdf]
