# Supplementary material for: Quantitative proteomics reveals TMOD1-related proteins associated with water balance regulation
Source: PLoS One. 2019 Jul 24;14(7):e0219932. doi: 10.1371/journal.pone.0219932 (PMC6656345; doi:10.1371/journal.pone.0219932)
Supplement: S1 Table — (DOC) [file pone.0219932.s002.doc]

**S1 Table. Significantly up-regulated proteins identified by LC-MS/MS.**

| **Majority protein IDs** | **Protein names** | **Gene names** | **iBAQ** | **Foldchange**  **(TFK/TF)** | **-Log *t*-test**  ***p*-value** |
| --- | --- | --- | --- | --- | --- |
| Q8R0G9 | Nuclear pore complex protein Nup133 | Nup133 | 7398400 | 2.07 | 1.88 |
| P58021 | Transmembrane 9 superfamily member 2 | Tm9sf2 | 466990000 | 2.07 | 2.18 |
| Q810A7 | ATP-dependent RNA helicase DDX42 | Ddx42 | 30786000 | 2.09 | 1.95 |
| G3X9T8 | Ceruloplasmin | Cp | 49606000 | 2.14 | 1.56 |
| Q8CI78 | Required for meiotic nuclear division protein 1 homolog | Rmnd1 | 75032000 | 2.16 | 1.37 |
| O88343 | Electrogenic sodium bicarbonate cotransporter 1 | Slc4a4 | 1204500000 | 2.23 | 1.54 |
| Q5FW60 | Major urinary protein 20 | Mup20 | 402540000 | 2.35 | 1.33 |
| Q8BFZ3 | Beta-actin-like protein 2 | Actbl2 | 404380000 | 2.62 | 1.44 |
| Q7TMR0 | Lysosomal Pro-X carboxypeptidase | Prcp | 78512000 | 2.71 | 1.40 |
| P06728 | Apolipoprotein A-IV | Apoa4 | 69982000 | 2.74 | 2.28 |
| A0A0A6YXD3 | NADH dehydrogenase [ubiquinone] iron-sulfur protein 2, mitochondrial | Ndufs2 | 3259400000 | 3.77 | 1.51 |
| Q7TQI3 | Ubiquitin thioesterase OTUB1 | Otub1 | 103000000 | 4.00 | 1.80 |
| Q99J94 | Solute carrier organic anion transporter family member 1A6 | Slco1a6 | 57779000 | 5.16 | 4.40 |
