# Supplementary material for: Quantitative proteomics reveals TMOD1-related proteins associated with water balance regulation
Source: PLoS One. 2019 Jul 24;14(7):e0219932. doi: 10.1371/journal.pone.0219932 (PMC6656345; doi:10.1371/journal.pone.0219932)
Supplement: S2 Table — (DOC) [file pone.0219932.s003.doc]

**S2 Table. Significantly down-regulated proteins identified by LC-MS/MS.**

| **Majority protein IDs** | **Protein names** | **Gene names** | **iBAQ** | **Foldchange**  **(TFK/TF)** | **-Log *t*-test**  ***p*-value** |
| --- | --- | --- | --- | --- | --- |
| A8Y5N8 | Pleckstrin homology domain-containing family F member 2 | Plekhf2 | 85803000 | 0.00 | 4.75 |
| Q3UU96 | Serine/threonine-protein kinase MRCK alpha | Cdc42bpa | 1523400 | 0.00 | 2.09 |
| D3YZ98 | Phosphatidylinositol 3-kinase catalytic subunit type 3 | Pik3c3 | 8678400 | 0.00 | 5.89 |
| D3Z5R2 | transmembrane protein 116 | Tmem116 | 147200000 | 0.00 | 4.53 |
| E0CYW7 | Hepatoma-derived growth factor | Hdgf | 77397000 | 0.00 | 5.25 |
| E9Q2A6 | Protein-tyrosine kinase 2-beta | Ptk2b | 3575000 | 0.00 | 2.92 |
| M0QWP3 | Glycine N-acyltransferase-like protein | Gm4952 | 36565000 | 0.00 | 1.77 |
| O54781 | SRSF protein kinase 2 | Srpk2 | 5238400 | 0.00 | 4.25 |
| P70698 | CTP synthase 1 | Ctps1 | 20685000 | 0.00 | 3.68 |
| P97770 | THUMP domain-containing protein 3 | Thumpd3 | 29495000 | 0.00 | 3.36 |
| Q3TIV5 | Zinc finger CCCH domain-containing protein 15 | Zc3h15 | 26990000 | 0.00 | 2.90 |
| Q3U0S6 | Ras-interacting protein 1 | Rasip1 | 6740800 | 0.00 | 2.94 |
| Q9JII5 | DAZ-associated protein 1 | Dazap1 | 81100000 | 0.00 | 2.66 |
| Q3UGC7 | Eukaryotic translation initiation factor 3 subunit J-A | Eif3j1;Eif3j2 | 76632000 | 0.00 | 2.42 |
| Q62312 | TGF-beta receptor type-2 | Tgfbr2 | 2924300 | 0.00 | 2.60 |
| Q6P9R2 | Serine/threonine-protein kinase OSR1 | Oxsr1 | 37358000 | 0.00 | 2.20 |
| Q8BH57 | WD repeat-containing protein 48 | Wdr48 | 3010200 | 0.00 | 2.75 |
| Q99KR8 | Plasma alpha-L-fucosidase | Fuca2 | 11651000 | 0.00 | 2.39 |
| Q9CRB8 | Mitochondrial fission process protein 1 | Mtfp1 | 53502000 | 0.00 | 2.82 |
| Q9D7X8 | Gamma-glutamylcyclotransferase | Ggct | 49576000 | 0.00 | 1.73 |
| Q9JLI8 | Squamous cell carcinoma antigen recognized by T-cells 3 | Sart3 | 47659000 | 0.00 | 4.56 |
| Q9Z329 | Inositol 1,4,5-trisphosphate receptor type 2 | Itpr2 | 4441400 | 0.00 | 2.83 |
| Q8VDI9 | Alpha-1,2-mannosyltransferase ALG9 | Alg9 | 16321000 | 0.08 | 1.92 |
| A0A087WPX1 | Aminoacylase-1 | Acy1 | 2095100000 | 0.11 | 1.78 |
| Q6PJN8 | DALR anticodon-binding domain-containing protein 3 | Dalrd3 | 6291700 | 0.12 | 2.41 |
| H3BKN0 | tRNA (cytosine(34)-C(5))-methyltransferase | Nsun2 | 24459000 | 0.13 | 2.15 |
| B0QZX9 | Dephospho-CoA kinase domain-containing protein | Dcakd | 169760000 | 0.14 | 1.55 |
| Q8R574 | Phosphoribosyl pyrophosphate synthase-associated protein 2 | Prpsap2 | 44492000 | 0.16 | 1.86 |
| Q9CQE3 | 28S ribosomal protein S17, mitochondrial | Mrps17 | 131450000 | 0.18 | 1.69 |
| Q64669 | NAD(P)H dehydrogenase [quinone] 1 | Nqo1 | 275740000 | 0.18 | 1.92 |
| O89103 | Complement component C1q receptor | Cd93 | 12681000 | 0.19 | 1.35 |
| Q9EQU5 | Protein SET | Set | 87507000 | 0.19 | 1.30 |
| P56376 | Acylphosphatase-1 | Acyp1 | 191900000 | 0.19 | 1.51 |
| Q0VBL3 | RNA-binding protein 15 | Rbm15 | 10383000 | 0.19 | 1.76 |
| Q99JX7 | Nuclear RNA export factor 1 | Nxf1 | 18687000 | 0.20 | 1.44 |
| Q3TZM9 | GDP-Man:Man(3)GlcNAc(2)-PP-Dol alpha-1,2-mannosyltransferase | Alg11 | 36337000 | 0.21 | 1.58 |
| P49183 | Deoxyribonuclease-1 | Dnase1 | 172040000 | 0.21 | 1.56 |
| G5E8J9 | SCY1-like protein 2 | Scyl2 | 18089000 | 0.21 | 1.35 |
| Q8C0V0 | Serine/threonine-protein kinase tousled-like 1 | Tlk1 | 5792500 | 0.21 | 1.32 |
| Q80X95 | Ras-related GTP-binding protein A | Rraga | 18828000 | 0.22 | 1.74 |
| Q9CYN9 | Renin receptor | Atp6ap2 | 31292000 | 0.22 | 1.57 |
| P54818 | Galactocerebrosidase | Galc | 66872000 | 0.22 | 1.49 |
| Q7TMG8 | Protein NipSnap homolog 2 | Gbas | 1003200000 | 0.22 | 1.53 |
| Q61191 | Host cell factor 1 | Hcfc1 | 21958000 | 0.22 | 1.32 |
| P03958 | Adenosine deaminase | Ada | 24874000 | 0.23 | 1.41 |
| Q9D832 | DnaJ homolog subfamily B member 4 | Dnajb4 | 51040000 | 0.23 | 1.54 |
| Q9DD02 | Protein Hikeshi | L7rn6;l7Rn6 | 196540000 | 0.23 | 1.36 |
| Q9ESJ0 | Exportin-4 | Xpo4 | 7682800 | 0.24 | 1.44 |
| P61082 | NEDD8-conjugating enzyme Ubc12 | Ube2m | 163080000 | 0.24 | 1.61 |
| Q9CZU6 | Citrate synthase, mitochondrial | Cs;Csl | 3384000000 | 0.25 | 1.87 |
| Q8K0C9 | GDP-mannose 4,6 dehydratase | Gmds | 54703000 | 0.25 | 1.37 |
| Q8BU88 | 39S ribosomal protein L22, mitochondrial | Mrpl22 | 36980000 | 0.26 | 1.33 |
| Q8VIM9 | Immunity-related GTPase family Q protein | Irgq | 4889900 | 0.26 | 1.32 |
| Q8BFR4 | N-acetylglucosamine-6-sulfatase | Gns | 59951000 | 0.29 | 1.41 |
| Q8K274 | Ketosamine-3-kinase | Fn3krp | 142340000 | 0.32 | 1.32 |
| Q99JB7 | Protein amnionless | Amn | 92833000 | 0.33 | 1.49 |
| Q9CXD6 | Mitochondrial calcium uniporter regulator 1 | Mcur1 | 139910000 | 0.34 | 1.31 |
| O55125 | Protein NipSnap homolog 1 | Nipsnap1 | 2962300000 | 0.35 | 1.49 |
| Q9CR62 | Mitochondrial 2-oxoglutarate/malate carrier protein | Slc25a11 | 482300000 | 0.36 | 1.32 |
| Q9CPR5 | 39S ribosomal protein L15, mitochondrial | Mrpl15 | 120810000 | 0.37 | 1.50 |
| Q78IK2 | Up-regulated during skeletal muscle growth protein 5 | Usmg5 | 2011800000 | 0.37 | 1.47 |
| Q9JLT2 | Trehalase | Treh | 7867200 | 0.37 | 1.40 |
| Q9CQI3 | Glia maturation factor beta | Gmfb | 327480000 | 0.38 | 1.85 |
| Q91WL5 | Cytochrome P450 4A12A | Cyp4a12a | 28750000 | 0.39 | 1.33 |
| Q8BLF1 | Neutral cholesterol ester hydrolase 1 | Nceh1 | 903800000 | 0.43 | 1.67 |
| Q9DBM2 | Peroxisomal bifunctional enzyme;Enoyl-CoA hydratase/3,2-trans-enoyl-CoA isomerase;3-hydroxyacyl-CoA dehydrogenase | Ehhadh | 10356000000 | 0.46 | 1.99 |
| P16125 | L-lactate dehydrogenase B chain;L-lactate dehydrogenase | Ldhb | 14204000000 | 0.46 | 1.66 |
| Q8R5L1 | Complement component 1 Q subcomponent-binding protein, mitochondrial | C1qbp | 1496200000 | 0.46 | 1.35 |
| P47740 | Fatty aldehyde dehydrogenase | Aldh3a2 | 3504300000 | 0.46 | 1.70 |
| P35564 | Calnexin | Canx | 774740000 | 0.51 | 1.97 |
