# Supplementary material for: Quantitative proteomics reveals TMOD1-related proteins associated with water balance regulation
Source: PLoS One. 2019 Jul 24;14(7):e0219932. doi: 10.1371/journal.pone.0219932 (PMC6656345; doi:10.1371/journal.pone.0219932)
Supplement: S3 Table — (DOC) [file pone.0219932.s004.doc]

**S3 Table. Details of the gene sets enriched by GSEA in TF group.**

| **Gene set name** | **Size** | **NES** | **NOM *p*-value** | **FDR *q*-value** |
| --- | --- | --- | --- | --- |
| KEGG_GNRH_SIGNALING_PATHWAY | 19 | -1.51 | 0.005 | 0.216 |
| KEGG_PHOSPHATIDYLINOSITOL_SIGNALING_SYSTEM | 15 | -1.50 | 0.014 | 0.130 |
| KEGG_CALCIUM_SIGNALING_PATHWAY | 23 | -1.40 | 0.014 | 0.311 |
| KEGG_OOCYTE_MEIOSIS | 26 | -1.38 | 0.021 | 0.285 |
| KEGG_GLUTATHIONE_METABOLISM | 30 | -1.31 | 0.022 | 0.401 |
| KEGG_UBIQUITIN_MEDIATED_PROTEOLYSIS | 29 | -1.29 | 0.042 | 0.426 |
| KEGG_VASCULAR_SMOOTH_MUSCLE_CONTRACTION | 23 | -1.29 | 0.048 | 0.397 |
| KEGG_CHEMOKINE_SIGNALING_PATHWAY | 31 | -1.27 | 0.050 | 0.416 |
